# TECHNOLOGY-RELATED COVID-19 EXPERIENCES OF OLDER VETERANS

**DOI:** 10.1093/geroni/igae098.2907

**Published:** 2024-12-31

**Authors:** Saanvi Lamba, Pranjal Tyagi, Diana Ruiz, Stuti Dang

**Affiliations:** Flint Hill School, Oakton, Virginia, United States; South Florida Veteran Affairs Foundation for Research and Education (SFVAFRE), Miami, Florida, United States; Miami Veterans Affairs Healthcare System, Miami, Florida, United States; Miami VA Healthcare System, Miami, Florida, United States

## Abstract

We fielded the HERO CARE survey to 20,000 community-dwelling Veterans from five Veterans Affairs’ (VA) Medical sites. Veterans were asked an open-ended question to “share anything else that you would like us to know about your experience with the COVID-19”. We received responses from 1,992 (95.8%) males and 88 (4.2%) female veterans; 23 responses were technology-related. Technology-related comments were made by 18 male respondents [79.2 + 10.2y on average, 66.6% non-Hispanic White (NHW)], and 5 female respondents [75.8±14.0y on average, 42.9% NHW, 42.9% non-Hispanic Black]. Three coders classified these through inductive coding into emergent themes. The most common themes were trouble with technology access, technology-related usability challenges, using technology for communication during isolation, using technology for healthcare/appointments, and an inability/preference to see providers in person. A positive impact was the technology use for staying connected during isolation, endorsed by the statements, “It has reduced my ability to attend church in person I now attend via Facebook Live.”, and “Have made more frequent use of the Internet and have communicated with family and friends worldwide.” Older Veterans used technology to connect to VA care and with friends and family, despite challenges with technology access and usability, and sometimes a preference for in-person visits. The COVID-19 pandemic accelerated the adoption and innovative uses of technology by older veterans to enhance communication, combat social isolation, and for healthcare visits. Assisting older adults with tools and training to address access and usability challenges may allow more equitable use of technology use for enhancing access and communication.

